# Passport to freedom? Immunity passports for COVID-19

**DOI:** 10.1136/medethics-2020-106365

**Published:** 2020-08-15

**Authors:** Rebecca C H Brown, Julian Savulescu, Bridget Williams, Dominic Wilkinson

**Affiliations:** 1 Oxford Uehiro Centre for Practical Ethics, University of Oxford, Oxford, UK; 2 School of Public Health and Preventive Medicine, Monash University, Clayton, Victoria, Australia

**Keywords:** public health ethics, public policy, ethics

## Abstract

The COVID-19 pandemic has led a number of countries to introduce restrictive ‘lockdown’ policies on their citizens in order to control infection spread. Immunity passports have been proposed as a way of easing the harms of such policies, and could be used in conjunction with other strategies for infection control. These passports would permit those who test positive for COVID-19 antibodies to return to some of their normal behaviours, such as travelling more freely and returning to work. The introduction of immunity passports raises a number of practical and ethical challenges. In this paper, we seek to review the challenges relating to various practical considerations, fairness issues, the risk to social cooperation and the impact on people’s civil liberties. We make tentative recommendations for the ethical introduction of immunity passports.

## Introduction

As the COVID-19 pandemic progresses, many people worldwide will contract the virus and recover. Many of these will be asymptomatic or experience mild symptoms only.^1^ Due to the novel nature of the virus, it is not yet clear what level of immunity is conferred by infection. However, evidence from COVID-19 thus far, and experience with previous coronaviruses, suggests that some level of protection from reinfection is likely in the short term, and may persist for several years.^2^ Thus, there is a good chance that people who have been infected and subsequently recovered are likely to be at least temporarily at lower risk of reinfection, less likely to suffer the harmful effects of the virus and less likely to spread the virus to other people.

Many countries, including the UK, have used strict lockdown measures in order to reduce the spread of the virus. These include social distancing (working from home and only leaving for essential purposes), school and university closures, mask wearing and home quarantining. Some of these measures result in enormous social and economic costs, severely restricting people’s interactions outside the home and preventing many from working. The UK government has sought to mitigate some of the economic harms by introducing the Coronavirus Job Retention Scheme, whereby employees are furloughed (placed on temporary leave) and the government pays them 80% of their wages (up to £2500 a month). In June, the cost of the scheme was estimated at £60 billion for the expected 8 months of its duration (though this could be further extended).^3^ One estimate of the global economic cost associated with COVID-19 is £4.7–£7.1 trillion.^4 5^

One of the reasons such lockdown measures are deemed necessary is the potential for asymptomatic infection and spreading of COVID-19. Estimates vary widely, but the number of people who do not experience symptoms when infected with COVID-19 seems likely to be around 40%.^6^ Research suggests that close to half of all transmission events occur before the onset of symptoms, and people are thought to be at their most infectious on or before the time of symptom onset.^7 8^ Viable virus has been detected from patients as early as 6 days before the onset of symptoms.^9^ This means, to prevent spreading of the disease, even those without any symptoms need to be contained.

The risk of death or hospitalisation from COVID-19 infection increases with age and according to the presence of other underlying health conditions including heart disease, diabetes and immunological problems.^10 11^ Efforts to reduce the spread of infection are, in part, aimed at reducing the numbers of severe cases, and avoiding a large peak of severe infections. Such a peak could overwhelm the healthcare system, particularly critical care services, making it unable to effectively provide care to both COVID-19 sufferers and others. The diminished ability of healthcare systems to provide non-pandemic-related care may already be affecting public health. For instance, the UK Office for National Statistics (ONS) has reported a significant rise in excess mortality not labelled as due to COVID-19 since the outbreak started. While some of these may result from undercounting of COVID-19 deaths, others may be due to people not receiving care for other conditions (including, perhaps, an unwillingness to attend hospitals because of perceived COVID-19 risk).^12^

Those at low *personal* risk from COVID-19 (ie, unlikely to suffer serious harms from infection), and who pose a low *social* risk (ie, unlikely to spread the disease to others) might reasonably be permitted more freedom than current lockdown measures allow. One group at low personal risk and who pose low social risk are those who have been infected by COVID-19 and recovered, and are now likely to have some level of immunity to the virus. It has been indicated by the UK Health and Social Care Secretary, Matt Hancock, that ‘immunity certificates’ might be provided to those who have recovered from the virus.^13 14^ This follows the ambition laid out in the UK government’s plan for scaling up testing programmes, which includes mass antibody testing (‘pillar 3’ of the five-pillar plan): ‘Antibody tests offer the hope that people who think they have had the disease will know they are immune and get back to life as normal.’^15^ Other countries, including Germany and Chile, may also be considering similar schemes.^16^

The introduction of some form of immunity passport scheme (see box 1) raises a number of ethical and practical problems, many of which have provoked significant debate.^17–21^ In the following sections, we summarise concerns relating to practical considerations, fairness, civil liberties and the pressure placed on social cooperation.

Box 1 **Overview of immunity passports**

**What are immunity passports?**

- Immunity passports are a way of recording that an individual is believed to have immunity to COVID-19 and is presumed unlikely to contract or spread the disease. They could take the form of a certificate, wristband, mobile-based app or other document.
- Possessing an immunity passport could grant people freedoms otherwise suspended during partial/full lockdown, such as travelling to work and socialising with people outside the home.
- At present, no vaccine is available for COVID-19, so immunity is presumed, in the main, to be acquired by infection and subsequent recovery. This would need to be established via testing at the time of infection and/or subsequent testing for antibodies.
- It is unknown for how long after infection people remain immune to COVID-19 so passports may need an expiry date, or people may need to be retested to confirm continued immunity.
- Immunity passports could be used in combination with other measures, such as widespread testing, and contact tracing for infected cases.

## Practical considerations

First, it is worth briefly discussing some of the practical considerations relating to immunity passports. This discussion will not be exhaustive, but is intended as indicative of the key factors to bear in mind in considering the introduction of immunity passports.

A significant limitation on the introduction of immunity passports is the need for a sufficiently reliable rapid test for COVID-19 antibodies.^22^ Antibody, or serology, tests identify whether or not someone has antibodies to COVID-19, thus indicating whether or not they have previously been infected with the virus, and whether or not they are likely to mount an immune response preventing reinfection if they encounter the virus again.^23^ As mentioned, it is unclear the extent and duration of immunity infection and recovery from COVID-19 will result in. The WHO has repeatedly stated that there is no evidence of lasting immunity in those recovered from COVID-19.^22 24 25^ This is only true on a very restrictive understanding of what counts as ‘evidence’. The only way to establish with certainty that people are immune for 1 year, 10 years or their whole lives would be to wait that long after infection and test their immunity. But this is unhelpful in the short term, and there are other ways of making predictions about COVID-19 immunity.

Patients who have recovered from their COVID-19 illness have been found to have neutralising antibodies, which inhibit virus growth.^26^ Whether all illness results in sufficient levels of neutralising antibodies to prevent against reinfection is still under investigation. However, experience with other coronaviruses (including viruses that cause mild illness as well as more serious diseases like severe acute respiratory syndrome (SARS) and Middle East respiratory syndrome (MERS)) suggests that antibody responses are likely to persist for at least a year and protect against reinfection at least in the short term.^27^ Antibody responses in SARS and MERS waned after 2–3 years, which might suggest immune passports should be time limited. Reports of individuals becoming reinfected with COVID-19 are likely to be cases where the individual falsely tested negative in the setting of prolonged viral shedding.^28^ Assuming that antibodies do indicate solid immunity, the difficulty lies in correctly identifying those antibodies in a way that permits testing to be scaled up significantly, without exceeding a tolerable level of false positives/negatives.

Someone who is identified as having antibodies to a particular disease—in this case, COVID-19—is described as seropositive. Those without are seronegative. Antibodies might not be identifiable in blood tests until days or weeks after illness has resolved in the infected individual.^23 29^ There is therefore a delay between an individual being infected with COVID-19 and them testing seropositive. A key feature of diagnostic tests is their sensitivity and specificity. Sensitivity refers to the test’s capacity to correctly identify those who have antibodies as seropositive. Specificity refers to the test’s capacity to correctly identify those who lack antibodies as seronegative. A test that is very sensitive will have a low false negative rate: there will be few people who have antibodies whom the test erroneously identifies as seronegative. A test with very high specificity will have a low false positive rate: there will be few people whom the test identifies as seropositive who in fact lack antibodies.

It is particularly difficult to develop a serological test for COVID-19 that has very high sensitivity and specificity. The test relies on producing a protein unique to COVID-19 which antibodies will bind to (if they are present in the person’s blood). COVID-19 is a coronavirus, like many common cold-causing viruses. There is a risk that, if the protein used in antibody tests for COVID-19 is too similar to proteins present in other coronaviruses, many false positives will result because people will have antibodies from infections with other coronaviruses.^23^ In the context of immunity passports, a false negative will mean that someone who is immune to COVID-19 will need to continue observing lockdown requirements, while a false positive could be more disruptive, indicating that someone is protected from infection when they are not. Such an individual could receive an immunity passport while still at risk of contracting and spreading the virus.

Different tests vary in their accuracy and the quality of the evidence we have about their accuracy. In particular, sensitivity of tests in the first week or two after infection may be low.^29 30^ The numbers of false positives and false negatives such tests produce (and how disruptive these errors are) will depend significantly on the baseline rates of infection (ie, whether or not people commonly have antibodies for COVID-19 in their bloodstream). If the rates of seropositivity are low, many of those identified as being immune will be false positives. Seroprevalence will vary greatly: in some cities it may be as high as a fifth, though elsewhere it will be much lower.^31 32^ Similarly, among healthcare professionals, seroprevalence is likely to be higher than among the general population.^33^ Low specificity tests combined with low seroprevalence will result in high numbers of false positives, while high specificity and high seroprevalence will result in far fewer.

The first rapid serological test for COVID-19 approved by the US Food and Drug Administration was called Cellex, and has a sensitivity of 94% and specificity of 96%^1^.^34 35^ The quality of tests is continually improving however, for instance, Public Health England has now approved for use a test by Roche that has at least 99.8% specificity.^36^ To illustrate what this means for false positives/negatives, assume 3%–5% of the UK population (67 million) has been infected (as estimated by Neil Ferguson in April 2020).^37^ If everyone is either susceptible or immune this would equate to around 64.3 million susceptible and 2.68 million immune people in the UK. Using the (less accurate) Cellex test would correctly identify 94% of the 2.68 million immune people as seropositive, but would also incorrectly identify 2.6 million of those who are, in fact, seronegative, as being immune. In other words, 50% of those identified as immune would actually not be immune. If, however, the more accurate Roche test were used, on a population with higher baseline immunity (say, London: population 9 million, seroprevalence around 18%^38^) then false positives drop significantly (only 0.2% of those lacking antibodies would be incorrectly judged as seropositive, which in London would be 14 760 people).

How disruptive is it to identify non-immune individuals as being immune? Health experts have stated that an unreliable test is worse than no test at all.^15^ Whether or not this is true depends on how the test is used and the context in which it is deployed. For instance, false positives could be less damaging if they occur in young, healthy individuals who are less likely to suffer severe infection, if effective contact tracing could be upscaled and if the spread of infection of the virus can be maintained at a low rate through other interventions. Further, the damage caused by false positives is only meaningful when compared with the damage caused by alternative policies such as complete lockdown, with their attendant opportunity costs. It is therefore not necessarily the case that a test with an imperfect level of sensitivity and specificity is worse than no test. For example, if we compared a policy of releasing from lockdown all members of the community (as is occurring in many places), and a policy of selectively releasing those people who have apparent immunity, the latter would lead to much slower viral spread, even if the test had relatively low specificity.

If there are low levels of immunity in the general population, a test may need high specificity and sensitivity to be informative.^2^ However, in populations where baseline immunity is higher the positive predictive value (likelihood that someone is seropositive, given a positive test result) will be higher. Serological testing could therefore target populations with predicted high baseline immunity, those for whom lockdown is most personally and socially damaging (for instance, healthcare workers) and those who are less likely to suffer a severe infection if they did contract the virus after receiving a false positive result (younger people with no health vulnerabilities). We also need to be sufficiently confident that serology testing is telling us something of value: specifically, that people with antibodies to COVID-19 are no longer at risk of contracting the virus, and significantly less likely to infect others.

Further logistical difficulties arise concerning how people will be tested. It has been mooted that antibody testing could take place at home, using a small blood sample from a finger prick. Samples could then either be processed at home or sent to a lab for analysis.^39^ At-home testing reduces the transmission risks associated with people travelling outside their home, but increases the difficulty of ensuring that tests are performed correctly and that analysis and interpretation is also correct. Alongside such quality control problems, the incentives to acquire immunity passports mean people may try to cheat the test and fake positive results. If at-home testing is not possible then the costs of testing and the time taken to perform tests will be increased.

Another consideration is who should be prioritised for testing. As mentioned, this should in part be guided by the need to avoid harmful misdiagnoses. But, given limited testing capacity, it will also be necessary to focus testing on groups whose immunity status it will be most valuable to know. It is likely that testing will be prioritised to healthcare staff and other key workers. During April 2020 around 35 000 National Health Service (NHS) staff were reported off work because they or someone in their household had COVID-19 symptoms.^40^ Lack of staffing places a limitation on the capacity of the NHS to treat COVID-19 (and other) patients, and thus informs the extent to which lockdown measures are necessary. This capacity could be increased by facilitating immune staff to return to work. Similarly, other key workers such as care home staff, police, supermarket staff, transport workers and others whose continued work is essential to social functioning, would likely be prioritised for antibody testing.^41^ The public good created by enabling these groups to move around more freely justifies their position at the front of the queue for antibody testing and immunity passports.^3^ 42 It is unclear how other groups should be prioritised relative to one another, and such decisions will need to be made if immunity passports are to be introduced.

## Fairness

Lockdown restrictions are experienced unevenly by different people. While some are able to easily work from home, others will have lost their jobs or been furloughed. According to a survey conducted by the ONS, 29% of businesses reported laying off staff in the short term, with the accommodation and food services sector, the administrative and support services sector, and the arts, entertainment and recreation sector being the worst affected.^43^ In addition, people’s social interactions are likely to be significantly disrupted, with face-to-face interactions significantly reduced. Some individuals will be more or less able to relocate their social lives online; some will feel the loss of in-person socialising more acutely than others. Those living with domestic abuse may be at greater risk of violence, with some evidence that deaths from domestic abuse have increased during the lockdown period.^44^

Immunity passports would create their own differential effects. If introduced, it is likely that immunity passport holders would be permitted freedoms such as increased travel outside the home, including travel to work. There is some concern that people are not seeking medical care for non-COVID-19 illnesses, for fear of contracting the virus in healthcare settings, or overburdening health services.^45^ Those tested and shown to be immune may be more willing to engage with healthcare and thus experience better health in the longer term. Since immunity passport holders would be assumed at low risk of contracting or spreading the disease, they may be permitted to visit others for socialising, or asked to support key services (such as volunteering in the health and social care sector) that are suffering from a loss of staff due to sickness and self-isolation. It is likely that possessing an immunity passport would be a significant benefit, advantaging holders relative to non-holders.

The disparity between the freedoms permitted to immunity passport holders versus non-holders could be deemed unfair. On a simple model of fairness, that requires all people to be treated the same in a strict sense, this would indeed be the case. We should, however, consider whether such a simplistic model of fairness is appropriate, and further, the extent to which we are willing to privilege fairness above other values (such as benefits to individual well-being and economic recovery).

First, the distinction in treatment of the immune/susceptible groups may not be unfair since it is not arbitrary. Instead it tracks a salient difference between people, namely the risk posed to themselves and others by their free movement. Imagine Chris and Patrick both want to attend a concert by their favourite band, but Chris has to care for his daughter that evening and so cannot make it. We would not say that, since Chris must stay at home, Patrick ought to do so as well. Neither does this seem unfair. On the contrary, it would seem unfair to force *Patrick* to miss out on the concert simply because Chris cannot attend.

Antibody tests track two relevant properties which ground differences in treatment. The first is probability of infecting others. Quarantine and isolation are both founded on probability of infecting others. Early on in the pandemic, they were used on the basis of symptoms of a viral infection, travel to a high-risk region or contact with a proven case. These are all markers of probability of infection. Antibodies are a similar marker of the risk of being a threat to others.

The second relevant property is the probability of falling ill and using limited health resources. The whole point of ‘flattening the curve’ is to reduce this. Selective isolation of the elderly and those with comorbidities is justified on this basis.^46 47^ Appropriately deployed antibody testing is arguably an even more robust measure of likelihood of falling ill.

It is worth considering whether the freedoms reinstated for the immune actively harm those still susceptible to the virus. There are clear reasons why the opposite might be true: if at least some people are permitted greater freedom of movement then more people will be able to work and produce the goods that come from that work, be that in caring professions, construction, education, retail, communications, and so on; to pay taxes which will help support those unable to work; to volunteer to support struggling essential services, or offer assistance to those who cannot leave their homes, making ‘shielding’ efforts more effective. The more people that are able to return to some of their normal functioning, the better for society as a whole. Due to practical challenges in implementing an immunity passport scheme (for instance, logistical difficulties in arranging testing and certification), passports might be prioritised or only provided to certain groups, such as key workers, whose capacity for free movement is most valuable to society as a whole.

On the other hand, there is a risk of resentment from those unable to work or socialise due to their continued susceptibility. This could manifest in a reduction in social cohesion and a loss in feelings of support and solidarity, which could be important in the longer term efforts to manage the pandemic, as speculated by some behavioural scientists. For instance, Robert West (a health psychologist) has commented: ‘There’s so much evidence on “in group” and “out group” work that, even when you set up arbitrary “in groups” and “out groups”, people become quite tribal.’^48^ In the same article, Adam Oliver (a behavioural economist) states: ‘The whole approach might also undermine the message that we are all in this together, which is crucial if we are going to get through this relatively quickly.’ Any such resentment could be exacerbated if there were suspicions that people who were not, in fact, immune were moving around more freely under the pretext of immunity. The effect of such resentment and lost social cohesion could cause people to experience negative emotions and friction in their relations with others. They could also weaken compliance with lockdown requirements and increase spread of COVID-19. But note that these concerns are speculative: it is not clear if such harms will materialise or to what extent they will have destructive effects. It must be borne in mind that any harms here must be traded off with the benefits of allowing (some) people to return to work and other activities.

The duration of lockdown restrictions could vary. Even after relaxation, second or third waves of viral spread could provoke further national or more localised lockdowns. Delays to the provision of effective treatments and vaccinations could result in longer periods of lockdown, increasing the disadvantages experienced by those without immunity passports. In discussing the impact of yellow fever in New Orleans during the early 19th century, Kathryn Olivarius describes how some of those ‘acclimated’ to the disease came to possess ‘immunocapital’: ‘socially acknowledged lifelong immunity to a highly lethal virus, providing access to previously inaccessible realms of economic, political and social power’.^49^ But far from being a great leveller, the benefits of (perceived) immunity to yellow fever were realised asymmetrically, with those already most powerful (rich, white) best placed to exploit its advantages. The current global COVID-19 pandemic is a different disease in a different context. Although social inequality persists, legal slavery has long since been abolished in most of the world (although forms of effective slavery undoubtedly persist), and greater protections exist for those unable to work or leave their homes. Nonetheless, lockdown could cause two groups of immune and susceptible to become increasingly cemented. Those with verifiable immunity may be preferentially hired for jobs while those lacking immunity languish at home. Those entering the pandemic with greater financial reserves will be more resilient to the hardships lockdown brings. But those with fewer resources, whose work depends on them leaving the home but whose lack of immunity means they cannot do so, will be most vulnerable.

It is difficult to confidently predict how COVID-19 immunity passports would interact with existing inequalities. There is evidence that, in the UK at least, ethnic minority groups suffer disproportionately from COVID-19.^50^ This could result from a number of factors, including demography (more black and minority ethnic people live in London, where the pandemic was initially most intense); higher rates of pre-existing conditions leading to more severe infections; or increased exposure to the virus due to working in high-risk professions. While higher rates of infection mean greater risk, it could also, of course, lead to higher rates of immunity, advantaging these groups under an immunity passport scheme. We should not accept such a conclusion uncritically, however. Other considerations may also make a difference here, for instance, if antibody testing is made available privately, and so those with greater financial resources are better able to acquire tests (and establish immunity), this would once again suggest that inequalities are exacerbated rather than mitigated. Access to testing should not, therefore, rely on personal wealth.

Regardless of how COVID-19 immunity interacts with existing inequalities, the solution to the hardships likely to befall those who lack immunity and are most hard hit by lockdowns is not to force everyone (including those presumed immune to COVID-19) to maintain strict social distancing, but to convert the benefits they accrue from their increased freedom of movement into support for the least well off. Immunity passports, and the freedoms they bring, must be accompanied by a redistribution of the resources they create. This can be used to mitigate the harms suffered by those in lockdown, with priority given to the most vulnerable in society.

### Responsibility

Related to fairness is responsibility. There might be some concerns that those most likely to acquire immunity (and thus, immunity passports) are those who have been least conscientious in following government guidelines. In contrast, those who have been reckless and failed to social distance are more likely to contract COVID-19 and thus more likely to acquire immunity. Immunity passports could end up rewarding the reckless or, worse, incentivising reckless behaviour. We will discuss the perverse incentives of immunity passports in the next section. For now we will consider whether we should be concerned that those who least deserve immunity passports may be the ones most likely to receive them.^4^

One way to avoid this worry would be to limit the disadvantage that accrues to those who behave conscientiously and successfully avoid getting COVID-19. As discussed above, those remaining in lockdown might automatically be advantaged by the free movement of others, if those others were to offer assistance (by collecting groceries, for instance) for those in lockdown. But compensation could be more active: prioritising those in lockdown for home deliveries of goods and provision of services; additional welfare support made available; future taxation breaks related to how long one spent in lockdown, and so on. Essentially, it could be ensured that those who remain in lockdown (due to ineligibility for immunity passports) are compensated for doing so. It is unlikely to be possible to fully offset the disadvantages some face, but to the extent that their compliance brings public benefits (in terms of reducing viral spread) they should be rewarded.

As well as rewarding conscientious behaviour directly, it might also be desirable to punish reckless behaviour. The Coronavirus Act 2020, fast-tracked through parliament in late March, gives the UK government the power to restrict gatherings and events and issue fines for non-compliance.^51^ While fines can, and have, been issued for breaches of legally enforceable lockdown requirements (such as leaving home without a ‘reasonable excuse’ or attending a public gathering), government guidance goes further than the law requires (this has led to some controversy around police over-reaching their powers).^52 53^

Current legislation therefore restricts how the police can punish those breaching lockdown guidance. Formal systems of punishment (ie, legal sanctions) are not the only punishments available, however. People can, and are, shamed for breaching lockdown guidance. Powerful social norms have quickly been established around the acceptability of leaving one’s house during lockdown, travelling to work, meeting with others, coughing in public, and so on. Moralisation in the public health context is controversial,^54^ but the stigmatisation of antisocial, risky behaviour might be justified in contexts where there is a clear risk of harm to others, and a moral obligation to refrain from exposing others to that risk. Such informal systems of punishment might be fostered by government messaging around COVID-19 in order to establish norms and encourage their enforcement. Care must be taken, however, since there is potential for misapplication. For instance, those who do have a reasonable excuse to travel outside the home might nonetheless experience stigma for doing so if it is not clear that they have such an excuse.

Another option would be to withhold immunity passports from those who acquire immunity through reckless behaviour. For example, public education campaigns could make people aware that if they are caught violating the rules they will be ineligible for immunity passports. While in theory this proposal might satisfy desires for responsibility and desert to be respected, it is likely to be unenforceable and perhaps unjustifiable. First, it will be challenging to determine retrospectively whether an individual with immunity acquired it through blameworthy behaviour. Second, if the justification for keeping people in lockdown (and severely restricting their ordinary rights to freedom) is the risk they pose to others, it looks unjust to restrict those freedoms to people who no longer pose such a risk. The withholding of an immunity passport could thus be too harsh a punishment (ie, the person will have their freedom hugely curtailed for a potentially small infraction, such as visiting a shop to buy a non-essential item, or visiting a friend or relative).

Ultimately, the measures taken to avoid rewarding reckless and punishing conscientious behaviour will need to reflect how costly it is to enforce punishments for the reckless, and how valuable it is to provide rewards for the conscientious. This value could be both instrumental and intrinsic: punishment and rewards can instrumentally influence behaviour to encourage compliance, but they may also have intrinsic retributive value.^55^

## Social cooperation

Social dilemmas, such as ‘tragedy of the commons’ situations (eg, common grazing land and fishing stocks), are where there is an incentive for each individual to exploit the resources as much as possible, even though the best outcome for the collective is to protect resources from exhaustion. Such incentives exist in the case of COVID-19, where at least some people’s narrow self-interest is best served if everyone else follows government guidelines while they themselves do not. This would achieve the benefits of lockdown in terms of reduced viral transmission and shortening the pandemic outbreak, while allowing them to avoid some of the harms of lockdown policies. The threat of defection by those who prefer to free-ride on others’ compliance with lockdown guidance means that disincentives may be needed. These can take the form of formal or informal punishments/rewards, as discussed above.

The introduction of immunity passports risks creating a means by which people can disguise their free riding, and a further incentive to do so. Perhaps most worryingly, it could incentivise intentional exposure to the virus, in order to acquire an immunity passport ‘legitimately’.^56^ Efforts to contain transmission of COVID-19 could be undermined if people begin moving around more freely with the intention of acquiring COVID-19 and (hopefully) recovering in order to obtain immunity, or if people fake immunity passports or acquire them on the black market. In order to prevent this behaviour, additional disincentives might be required (perhaps requiring further new legislation), or more intrusive policing, all at additional cost. Harsh punishments might be introduced: for instance, it could be decided that those who deliberately become infected should be deprioritised for treatment, though this is likely to be considered overly punitive (the use of responsibility in allocative decisions is already controversial and rarely explicitly used^57 58^). Alternatively, other disincentives, such as fines, could be employed.

The possibility of fraudulent immunity passports means systems of verification will be needed: creating passports that are hard to forge, punishments for creating fake passports and trading them on the black market. Some of the economic benefits of immunity passports could, therefore, be lost in the costs associated with policing the validity and use of such passports. This is unfortunate, but it will ultimately depend on whether the costs associated with regulating an immunity passport scheme outweigh the benefits. The possibility of some free riding might be tolerable, depending on the level of compliance with lockdown required in order to control the pandemic. Distaste for free riders is common and a desire to avoid situations where defection is possible and to ensure that free riders are punished is understandable.^59 60^ If these feelings of suspicion and resentment translate into socially destructive non-cooperation the capacity to effectively contain the pandemic spread will be diminished. However, we should be clear-sighted in evaluating the harms of perverse incentives and free riding. At least some research suggests the likelihood of people pursuing intentional infection is small: a survey found that a large majority of people stated they would not consider intentional infection in order to acquire an immunity passport.^61^ If the likelihood of intentional infection is small or unlikely to contribute much to spread, and the harm of free riding is similarly unlikely to be significant, then our intuitive desire to ensure defectors do not benefit should be set aside in favour of the potential benefits of facilitating free movement for the immune, and the trickle-down benefits for the economy and rest of society.

A different approach to solving the problem of free riders might be a system of controlled intentional infection. There has been some discussion of the possibility of variolation—intentionally infecting people with COVID-19 under controlled conditions in order for them to develop an infection and subsequent immunity.^62–64^ This is the behaviour parents engage in when taking their children to ‘chickenpox parties’. In the case of COVID-19, it is likely to be extremely controversial and does not appear to have been considered at all by those working in public health, epidemiology or policy. Yet for those at very low risk of severe infection, the expected harms of infection could be smaller than those associated with lockdown. It will be challenging to ensure low risks, both for the individual and for society, are maintained throughout variolation. To protect the health of the population, a system whereby people are isolated from the very start of their illness and monitored throughout, might minimise the risk of transmission and prevent delayed presentations in those who unexpectedly need hospital care despite an absence of risk factors for severe disease. This would clearly depend on the effectiveness of the controlled circumstances under which variolation takes place, which could include, for instance, using a low infecting dose; screening the individual for any risk-heightening underlying conditions; setting up quarantine conditions ahead of time; and ensuring access to medical care if it is needed. If the virus proves too difficult to control without severe restrictions, and in the absence of a vaccine, it may be the case that most people will become infected with the virus anyway. Doing so under supervised, controlled conditions that ensure appropriate isolation might be preferable, particularly for those at high risk of exposure in their daily life (including healthcare and transport workers).

## Civil liberties

Widespread antibody testing could facilitate a faster return to a more normal way of life for at least some of the population. But it could also create new pressures to intrude on people’s privacy and free choice. One possibility is that people could be pressured to undergo testing in someone else’s interest (for instance, their employer). Employers may want all their employees to undergo testing in the hope that many of them acquire immunity passports and can return to work. Some have argued that it would be inappropriate to pressure people into testing in this manner.^65^ Further, even if an individual was found to have immunity (and be eligible for an immunity passport), they may feel uncomfortable returning to work, perhaps for fear that they may not have prolonged immunity, or that they might still be able to transmit the virus to those they live with.

The conduct of employers will be hard to monitor, and it is likely that some people will experience pressure to undergo testing, and share the results of testing, in order to return to work. It is worth considering whether antibody testing should be enforceable by the state, and/or if employers should be able to make demands regarding the testing of their employees. For instance, employers might insist that staff must test positive for antibodies in order to return to work. For those unable to work from home, this could create a choice between returning to work and risking unemployment. Additional legal protections may need to be put in place to protect those unable to work in such scenarios. Given the threat COVID-19 poses to others’ health, and the capacity for businesses to operate, it might be a reasonable step for employers to ensure that employees are tested, in the context of returning to work. This could be done in concert with making flexible working arrangements where possible (eg, if it is not necessary for people to be physically present in work, and they prefer not to be tested or not to trust the result of a test), as well as ensuring that informed consent procedures are adhered to, with particular reference to information regarding how test results will be shared.

Immunity passports are unlikely to be used in isolation, and it is more probable that they will form part of a collective set of practices aimed at a phased reduction in lockdown restrictions. One option is mass community testing, and the use of contact tracing apps. These would involve performing tens of thousands of swab tests (which identify whether or not someone is currently infected with the virus) a day, quarantining those who test positive.^66^ In addition, mobile phone apps could instantly alert anyone who has been in close physical contact to people who test positive for COVID-19 and instruct them to quarantine.^67^ The main drawbacks of such an approach are the feasibility associated with the high uptake required in order for mobile contact tracing to be effective, and the concerns raised by having large quantities of data about people’s precise movements collected and stored.^68^ Both the technological challenges and ethical concerns continue to be addressed.^69^

Interventions to contain the spread of COVID-19 can conflict with civil liberties and put pressure on safeguards to protect individuals’ privacy and medical data. It must, however, be recognised that the pandemic represents an extraordinary situation where the stakes are high, in the form of many thousands of lives being placed at risk directly from the virus, and many more indirectly. While restrictions of civil liberties (such as freedom of movement) and the diminished protection of others (such as privacy surrounding health information) should not be taken lightly, this must always be weighed against the importance of restoring other civil liberties, such as allowing some people to return to work, or to move around more freely. In addition, measures such as immunity passports, mass community testing and contract tracing could begin to lessen the economic damage of current lockdown measures, while still protecting the most vulnerable in society.

## Concluding remarks

If widespread lockdowns to combat the spread of COVID-19 continue to be used, immunity passports might provide a way of allowing individuals to recover some normality in the form of moving around more freely and returning to work. This has benefits to the individual and society more broadly, although the extent of these benefits could depend on baseline immunity levels. But introducing and regulating immunity passports brings with it a set of ethical and practical challenges. We have summarised some of these challenges relating to practical considerations, fairness, the restriction of civil liberties and pressure on social cooperation.

Surveying the ethical worries, it seems to us that, while we should take these concerns seriously, they are not insurmountable. A great deal rests on the availability of suitably accurate serological tests, and the capacity to establish with sufficient confidence that those with antibodies to COVID-19 will be immune from reinfection. The standards of evidence and accuracy here must not be *over*demanding, so as to miss the benefits antibody testing and immunity passports could deliver. The harms of inaccurate testing must be balanced against the harms (both economic, health, and social) of alternative policies such as full lockdown or relaxation of lockdown without testing.

If immunity passports can feasibly be provided without risking infection control, they should be used. In doing so we make a number of recommendations (summarised in box 2). Testing (and immunity passport provision) should be targeted to minimise harm and maximise benefits. To increase accuracy, they should be used where high baseline immunity is expected. Attention should be paid to the interaction of immunity passports with existing inequalities, and efforts made to ensure that the least well off, in particular, are protected from the disadvantages of lacking immunity. A number of areas require further research to establish the seriousness of their implications, including risks related to undermining social cohesion, and the risks of intentional infection. While caution is wise, it is also essential to balance it with the opportunity costs (and harms) that could result from *not* using tools such as immunity passports.

Box 2 **Key recommendations for the ethical introduction of immunity passports**

**Summary of key ethical recommendations**

- Minimise risk of harm: test those whose personal risk of infection is low (younger people with no underlying health conditions)
- Maximise potential benefit: prioritise testing for those whose freedom is most personally and socially beneficial (eg, care workers, those who cannot work from home).
- Weigh the damage of false positives (and negatives) with damage from alternative policies (eg, continued full lockdown, general easing of lockdown), including economic, social and health impacts.
- Attend to the risks of undermining social cohesion for infection control and well-being; conduct further research and track developing evidence.
- Facilitate free testing so access not limited by personal wealth.
- Ensure redistribution of benefits created by immunity passports; provide additional support to ease costs to those remaining in lockdown and avoid incentivising non-compliant behaviour.
- Consider introducing additional punishments for non-compliant behaviour; balance these with the expected harms of such behaviour and ensure they are proportionate to the risks they aim to discourage.
- Produce guidance regarding the use of antibody testing/immunity passports by employers.

## Footnotes

Strictly speaking these describe the ‘positive percent agreement’ and ‘negative percent agreement’, rather than sensitivity and specificity. This is because there is currently no reference standard available against which the accuracy of the test can be measured (ie, the accuracy of the Cellex test is judged based on its ability to identify ‘true’ positives and negatives, but our judgement of the ‘truth’ here is based on other tests and clinical judgements which could themselves be inaccurate).^30^

If population infection rates are much higher than the 4% estimate used here, as some argue could be the case (Lourenço, Paton *et al* 2020),^70^ false positive numbers would drop.

What counts contributing to the ‘public good’ is reasonably contentious (see eg 42). Without specifying the full parameters of the concept here, we intend to refer to reasonably uncontroversial benefits such as allowing essential workers to safely carry out their jobs.

This sets aside the fact that those exposed via, for example, high-risk (and essential) work will also be more likely to become infected and acquire immunity, with no suggestion that their behaviour be considered reckless.
